# Limited benefit of additional contrast-enhanced CT to end-of-treatment PET/CT evaluation in patients with follicular lymphoma

**DOI:** 10.1038/s41598-021-98081-x

**Published:** 2021-09-16

**Authors:** Gaetano Paone, Mariana Raditchkova-Sarnelli, Teresa Ruberto-Macchi, Marco Cuzzocrea, Emanuele Zucca, Luca Ceriani, Luca Giovanella

**Affiliations:** 1 Clinic for Nuclear Medicine and Molecular Imaging, Imaging Institute of Southern Switzerland, Ente Ospedaliero Cantonale, Bellinzona, Switzerland; 2 Medical Oncology, Oncology Institute of Southern Switzerland, Ente Ospedaliero Cantonale, Bellinzona, Switzerland; 3 Institute of Oncology Research and Faculty of Biomedical Sciences, Università della Svizzera Italiana, Bellinzona, Switzerland; 4 Clinic for Nuclear Medicine, University Hospital and University of Zurich, Zurich, Switzerland

**Keywords:** Nanoscience and technology, Oncology, Cancer, Medical research, Outcomes research, Cancer, Cancer imaging, Cancer metabolism, Haematological cancer

## Abstract

Despite follicular lymphoma (FL) is frequently characterized by a moderate increase of glucose metabolism, PET/CT examinations provides valuable information for staging and response assessment of the disease. The aim of the study was to assess and compare the diagnostic performance of PET/ldCT and PET/ceCT, respectively, in evaluating FL patients at the end of treatment. Fifty FL consecutive patients who underwent end-of-therapy PET/CT with both ldCT and ceCT were analyzed. Two blinded observers independently assessed PET/ldCT and PET/ceCT applying the Deauville score (DS) and Lugano classification criteria. PET imaging obtained after the end-of-treatment (EoT) was classified as showing PET and ce-CT matched response (concordant imaging group, CIG) or PET and ce-CT unmatched response (discordant imaging group, DIG). Relapse rate and Event-Free Survival (EFS) were compared between CIG and DIG patients. Overall, no differences in metabolic response classification were observed between PET/ldCT and PET/ceCT. In 13 (26%) patients PET/ceCT identified additional FDG-negative nodal lesions in mesenteric, retroperitoneal and iliac regions. However, in all cases, final DS remained unchanged and the additional results did not modify the following therapeutic decision. Among patients, who obtained complete metabolic response a comparable rate of relapse was registered in DIG 3/13 (23%) and CIG subgroups 5/20 (25%) [p = 0.899]. In all 3 DIG cohort patients who relapsed the recurrent disease involved also, but not exclusively, PET negative lymph nodes detected by ceCT. In overall population metabolic response defined by PET/ldCT predicted EFS [76% (group of patients with metabolic response) vs 35% (group of patients with residual disease), p = 0.0013] significantly better than ceCT-Based response assessment [75% (group of patients with complete response) vs 53% (group of patients with residual disease), p = 0.06]. Our study demonstrates a negligible diagnostic and predictive value of ceCT performed in addition to standard ^18^FDG PET/ldCT for EoT response evaluation in FLs. PET/ldCT should be performed as first-line imaging procedure, also in patients with prevalent abdominal and pelvic involvement, limiting the acquisition of ceCT in selected cases. This tailored approach would contribute to avoid useless radiation exposure and preserve renal function of patients.

## Introduction

Follicular lymphoma (FL) represents more than 20% of all low-grade or indolent non-Hodgkin lymphomas (i-NHL) and is characterized by slow tumor growth and, frequently, residual disease after treatment^1^. Generally, watchful waiting (WW) is considered a reasonable option in most i-NHLs^2,3^. When needed, available treatments aim to delete the onset of symptoms related to mass-effects while preserving the patients’ quality of life^4,5^. The risk of transformation to more aggressive phenotypes is about 20–30% after ten years and it has been associated with a worse prognosis^6,7^. Then, a reliable imaging method is warranted to assess patients with FL after treatment and address appropriate clinical actions. ^18^F-fluorodeoxyglucose (FDG) positron emission tomography/computed tomography (PET/CT) is the gold standard to stage, restage and monitor patients^8–10^. As previously reported, PET/CT is more sensitive and specific than contrast-enhanced CT (ceCT) for detecting post-therapy residual disease for both aggressive and indolent follicular lymphoma^11–13^. Accordingly, performing PET with contrast-enhanced CT (PET/ceCT), is debatable even if suggested by some authors for residual disease assessment in clinical practice^14,15^. The association of ceCT to PET/CT in FL may results in an improvement of the evaluation of patients with prevalent abdominal and pelvic involvement, due to could the increased anatomical definition of these anatomical region determined by contrast medium administration. This potential advantage may be more relevant in particular after the end of treatment in detecting residual lesions with low metabolic rate. Nevertheless, this hypothesis has not been investigated yet in literature. This lack of data could be partially explained by the wide range of treatments available for FL including chemo-immunotherapy, antibody treatment alone, radiotherapy and watchful waiting strategy. On the other hand, the introduction of an additional ceCT needs also to consider the risk of potential adverse effects and the increase of radiation exposure and health care costs.

Then, the aim of our exploratory study is to compare the diagnostic performance of PET/CT with either conventional unenhanced low-dose CT (PET/ldCT) or ceCT (PET/ceCT), to assess end of treatment (EoT) disease response in a group of not previously treated FL patients who received standard immune-chemotherapy as front line treatment.

## Results

The clinical characteristics of enrolled patients are summarized in Table 1. Among 50 patients 14(28%) had Grade 1, 15 (30%) Grade 2 and 21 (42%) Grade 3 FL.

**Table 1:** Patient characteristic.

Variable	Data
No. men	31
No. women	19
Median age (y)	64 (45–83)
Grading G1	14 (28%)
Grading G2	15 (30%)
Grading G3	21 (42%)
R-CHOP	27 (54%)
R-Benda	20 (40%)
R-Chlorambucil	3 (6%)
FLIPI 1	10
FLIPI 2	27
FLIPI ≥ 3	13

**Figure 1:**
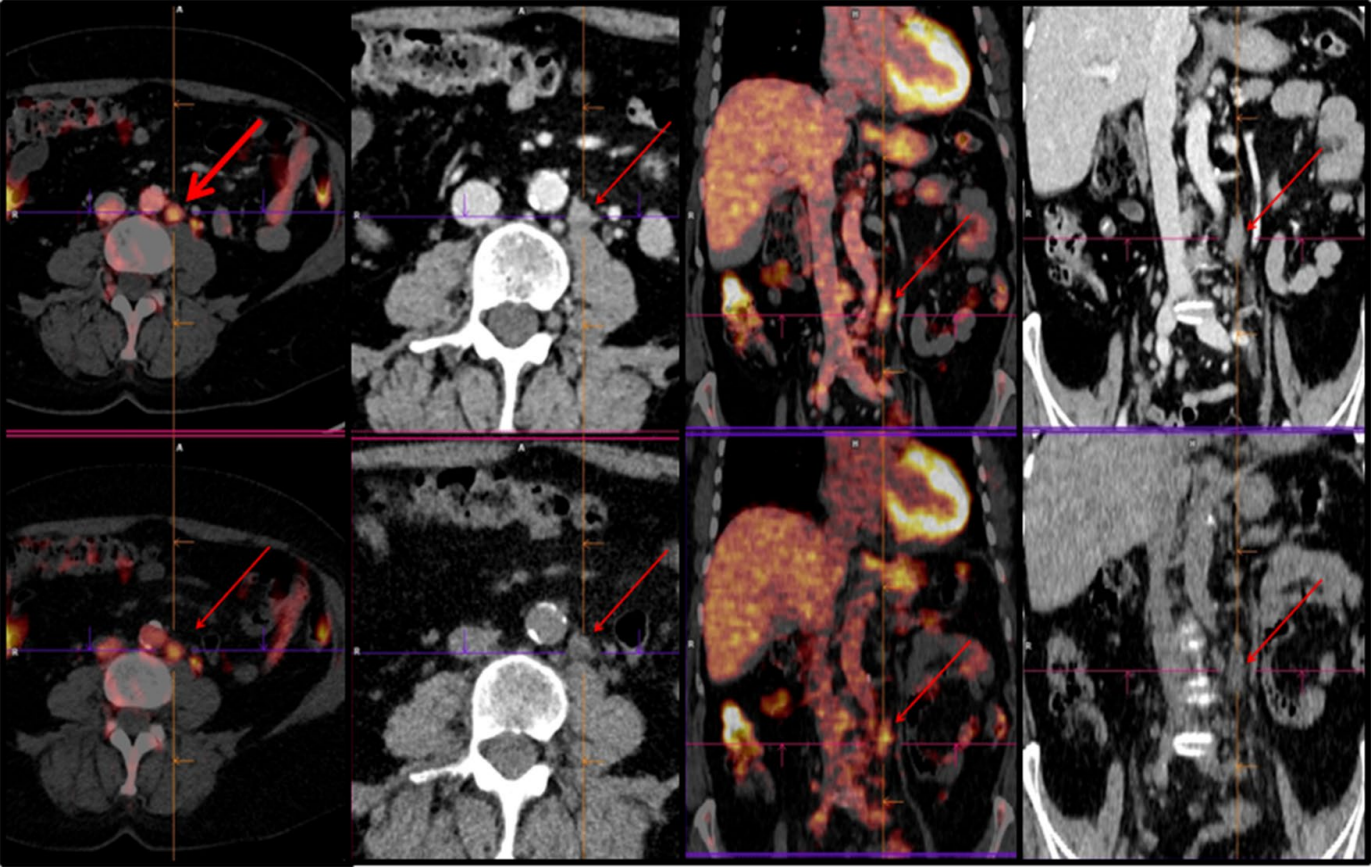
PET/ldCT showed the same number and sites of lesions highlighted by PET/ceCT [DS 5; SUVmax 8].

**Figure 2:**
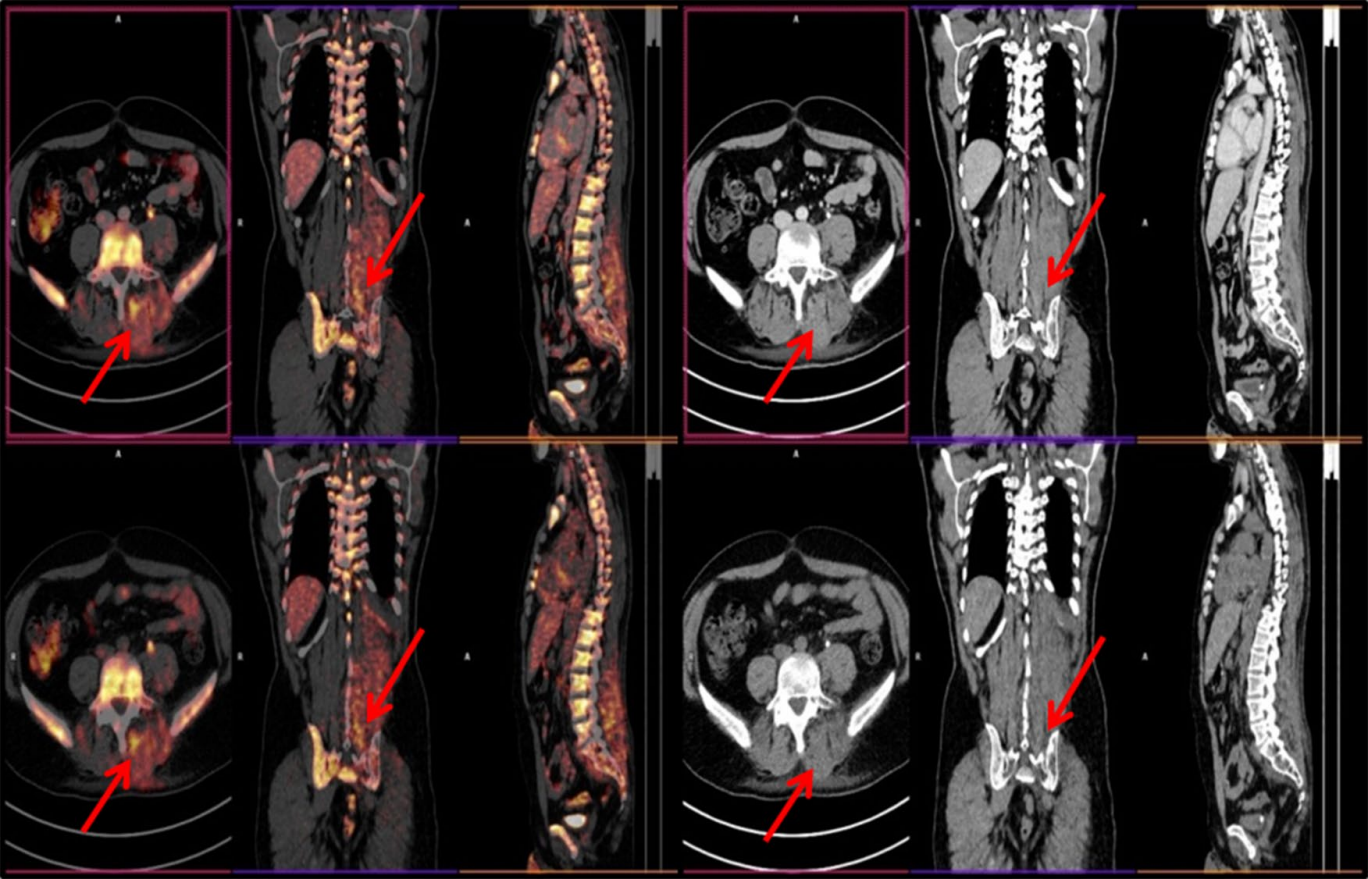
PET/ldCT and PET/ceCT showed paravertebral tissue infiltration [DS 5; SUVmax 4.7].

**Figure 3:**
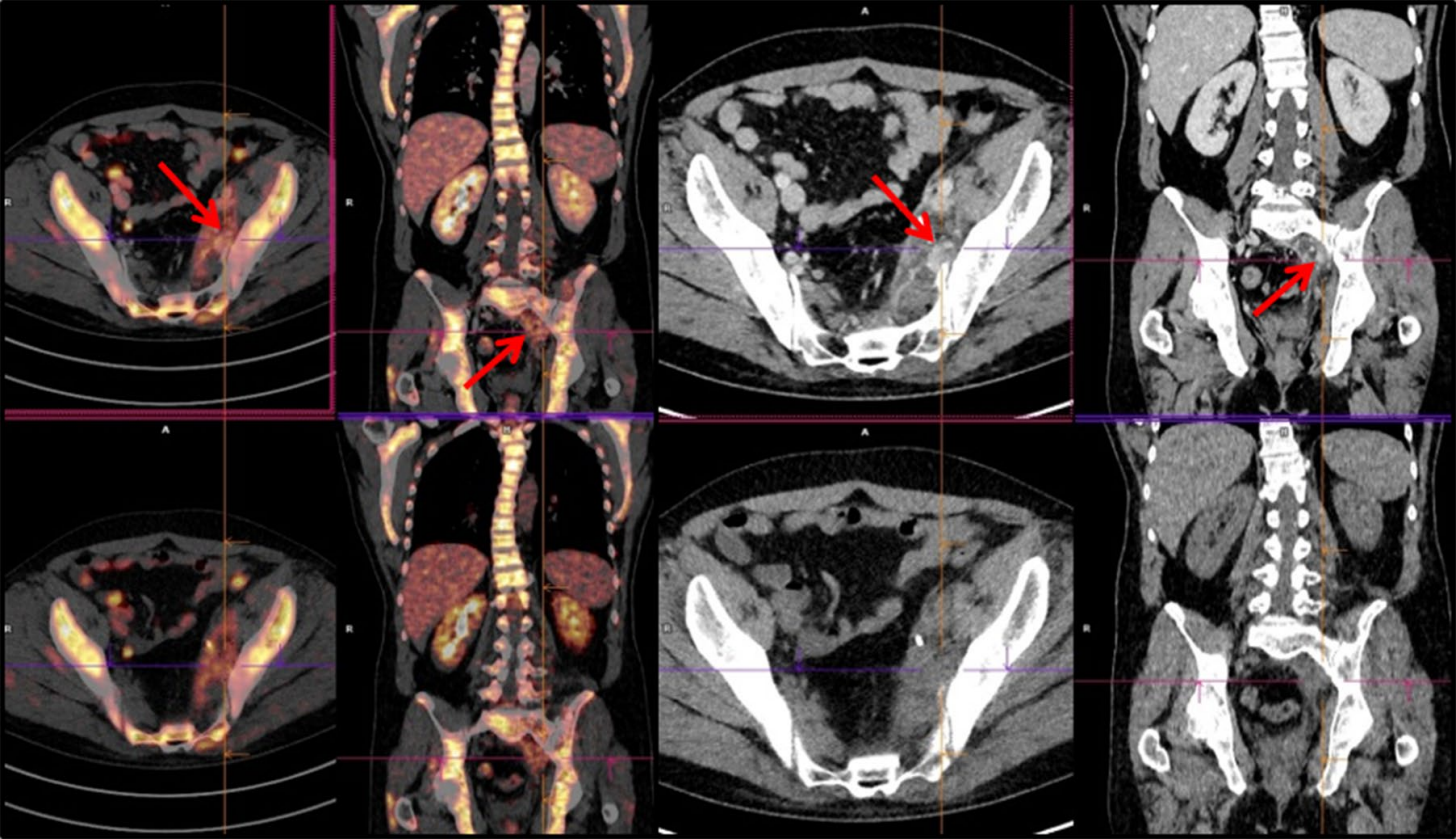
PET/ceCT showed additional nodal lesions in the left iliac region [DS 3; SUVmax 2.3].

**Figure 4:**
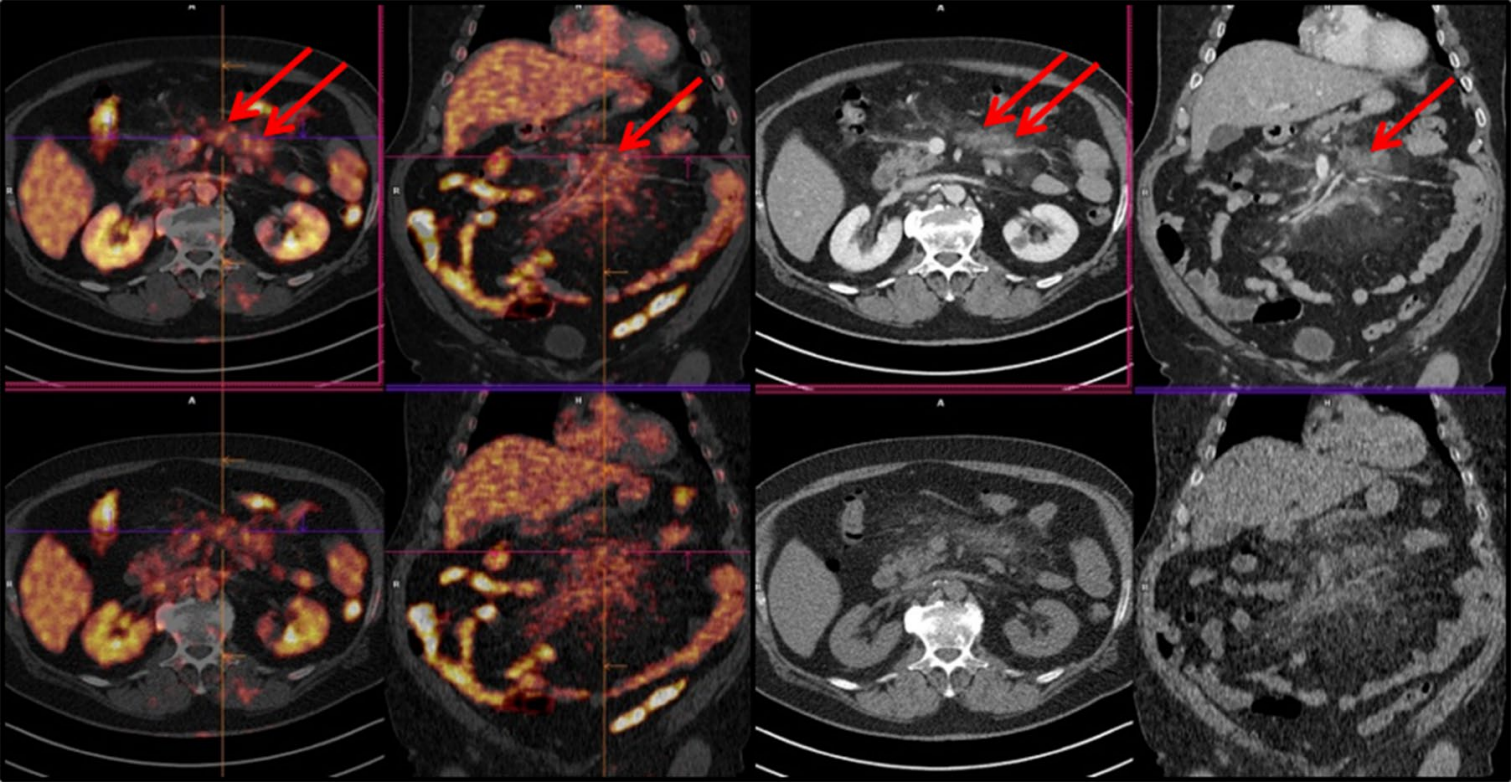
PET/ceCT highlighted additional lesions in mesenteric area [DS 4; SUVmax 4.1].

Concordant PET/ldCT and ceCT response assessment (CIG group) was observed in 37 of 50 patients (74%), 20 showing remission and 17 with residual or progressive disease (Table 2, Figs. 1, 2). In the remaining 13 (26%) PET negative patients there was discordant response classification (DIG) since ceCT identified 31 additional lymphadenopathies in mesenteric (n = 12), retroperitoneal (n = 9) and iliac regions (n = 10) (Chi square test p = 0.0245) (Figs. 3, 4). All the additional enlarged lymph nodes were reported as FDG-negative (i.e. uptake less than liver activity, DS ≤ 3) and, although the final DS changed in 2 patients from score 1 to score 2, the final metabolic response defined by PET/ldC was confirmed in all cases. Consequently, the following therapeutic decision based on PET results remained unchanged. On the other hand, in CIG group no difference in the number of residual lesions detected by PET-ldCT and ceCT was showed.

**Table 2:** EoT response in agreement with PET-based criteria.

Deauville score	CIG	DIG
CR	20	13
PR	7	–
PD	10	–

**Figure 5:**
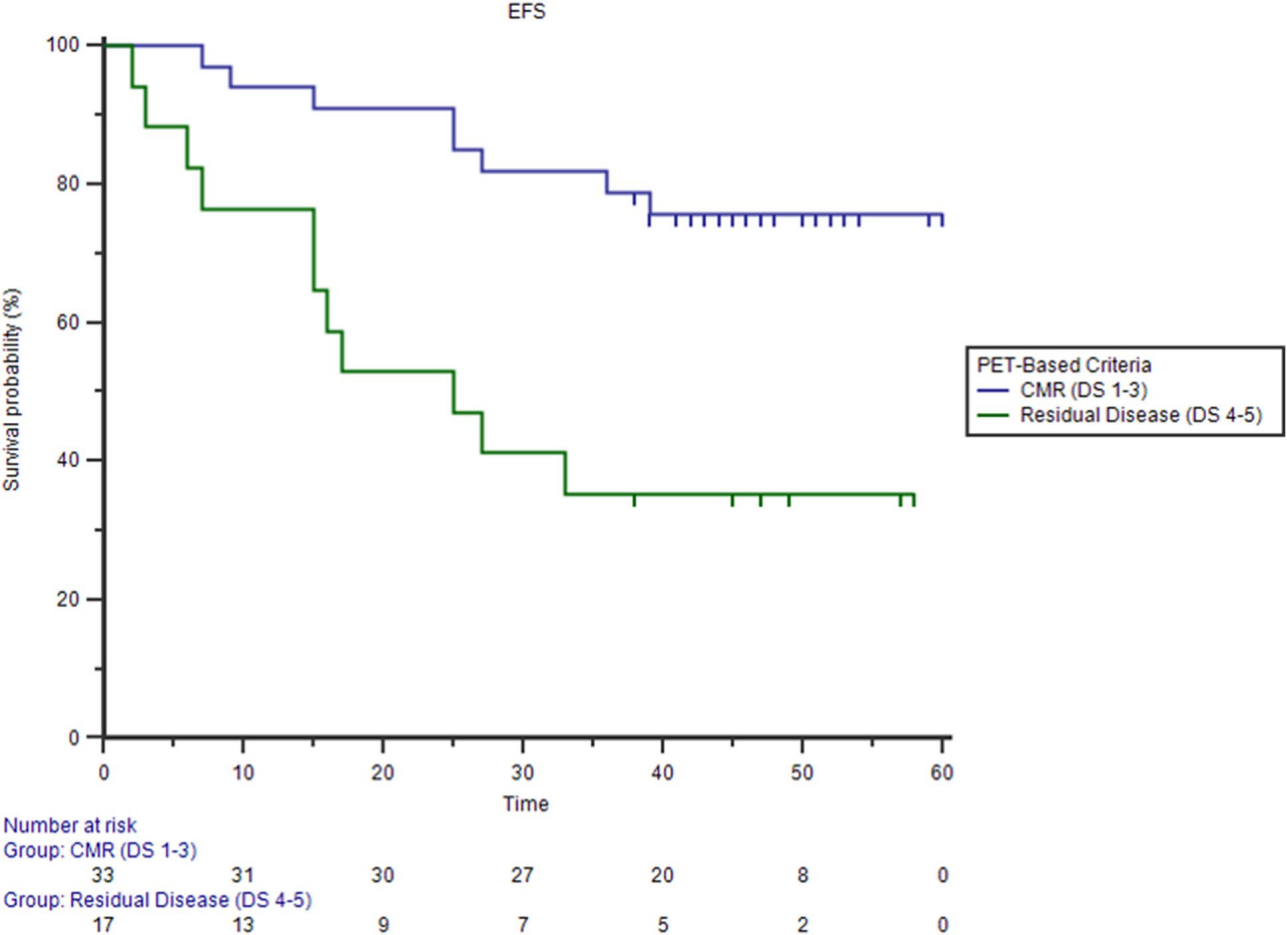
Kaplan–Meier estimates of event free survival (EFS) using PET-based criteria (76% vs 35% Logrank test p = 0.0013).

**Figure 6:**
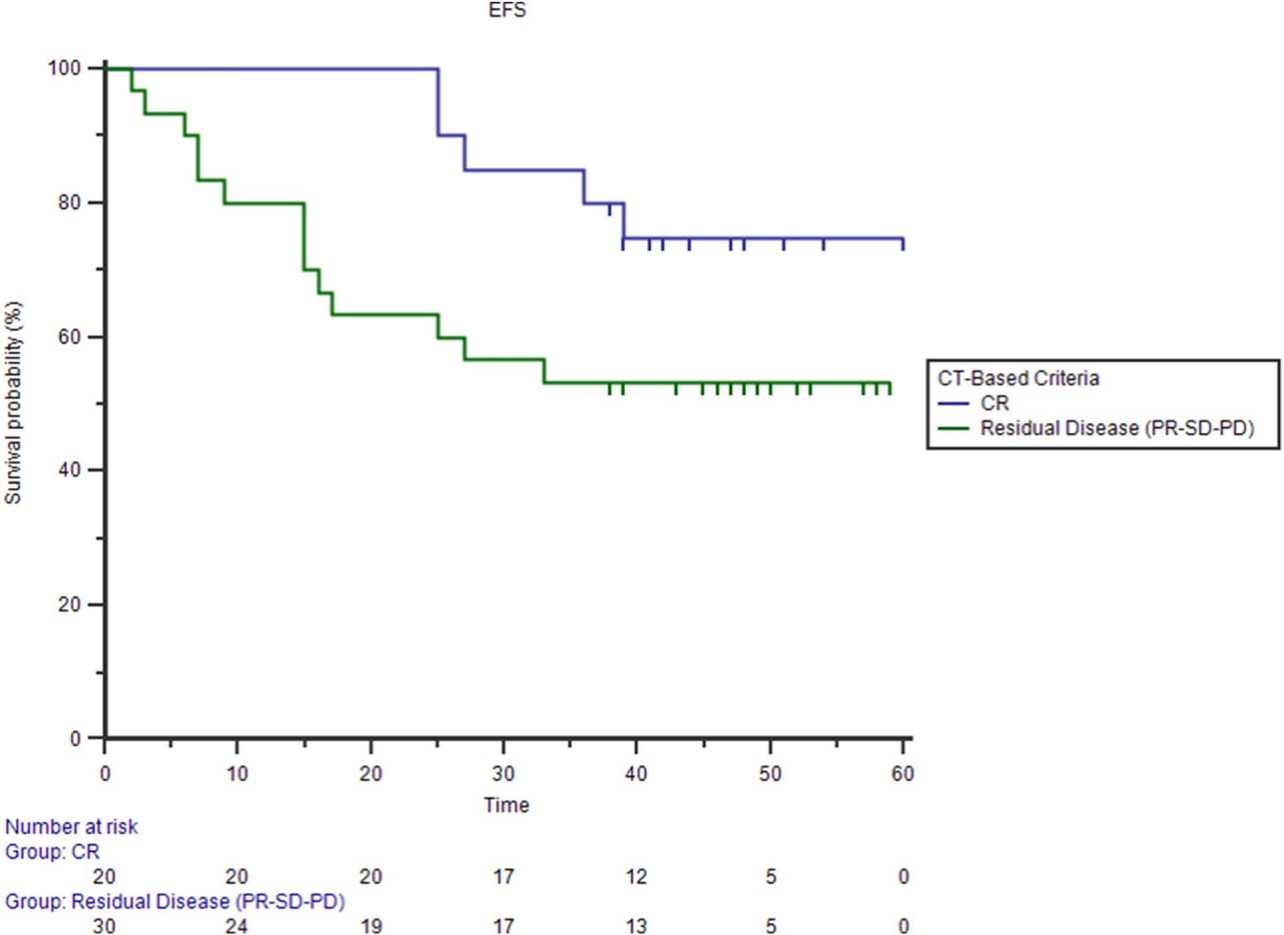
Kaplan–Meier estimates of event free survival (EFS) using CT-based criteria (75% vs 53% Logrank test p = 0.06).

Persistent or relapsed disease was registered in 19 patients (38%) after a follow-up of 36.5 ± 17.1 months. Among patients who obtained complete metabolic response (CMR) a comparable rate of relapse was registered in DIG 3/13 (23%) and CIG sub groups 5/20 (25%) [Chi square test p = 0.899]. In all 3 DIG cohort patients who relapsed the recurrent disease involved also, but not exclusively, PET negative lymph nodes detected by ce-CT. In overall population residual disease defined by PET/ldCT at EoT predicted poorer EFS (35% group with residual disease vs 76% group with metabolic response, Logrank test p = 0.0013; HR 3.8927, 95% CI 1.3983–10.8366) more accurately than ceCT-Based response assessment (53% group with residual disease vs 75% group with complete response, Logrank test p = 0.06; HR 2.4806; 95% CI 1.0076–6.1072) (Figs. 5, 6).

Positive predictive value (PPV) and negative predictive value (NPV) were 65%, 76% and 46%, 75% for PET/ldCt and ceCT assessment, respectively.

**Figure 7:**
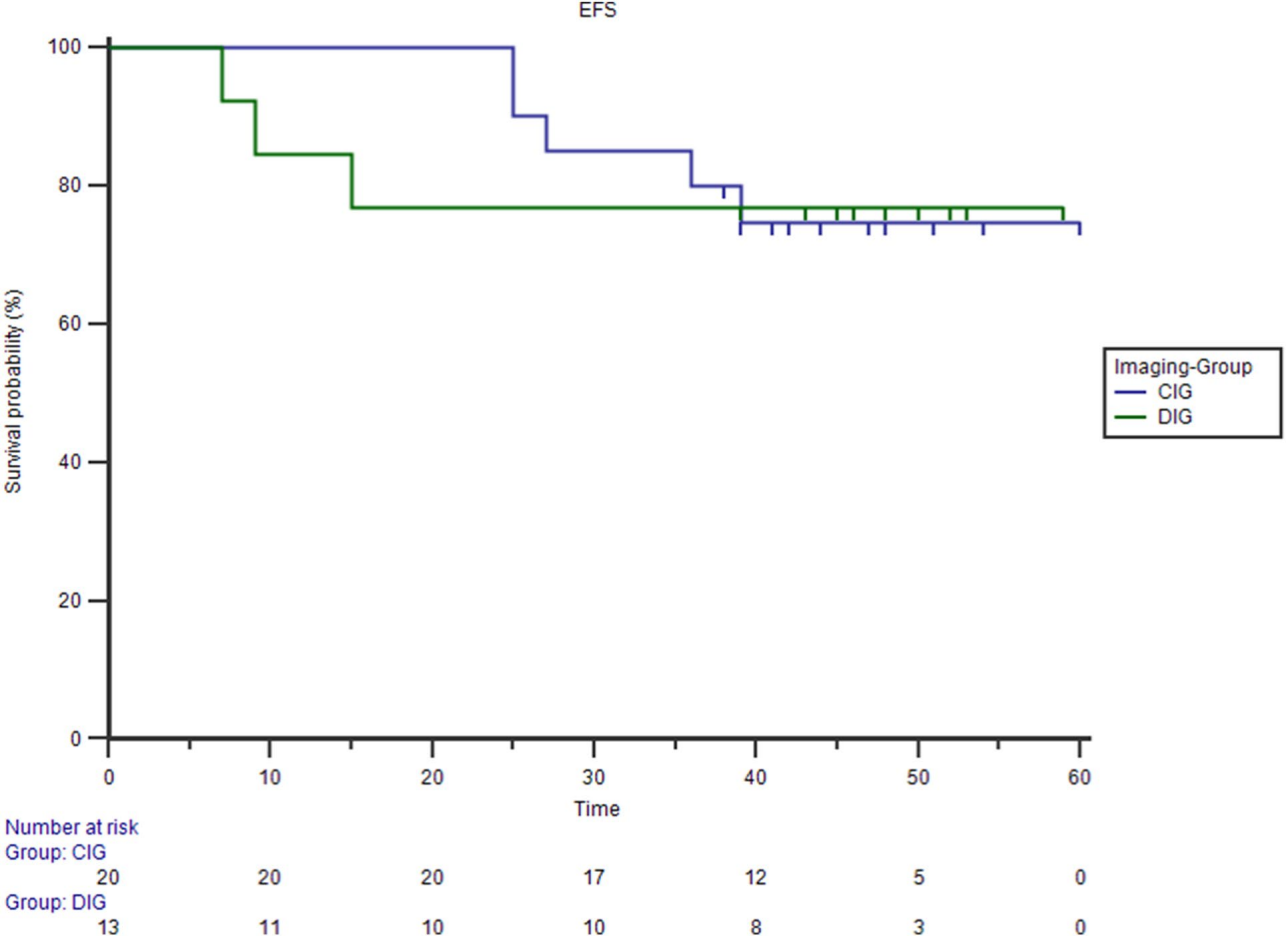
Kaplan–Meier estimates of event free survival (EFS) between CIG and DIG. No different EFS was detect between CIG and DIG patients obtained CMR (75% vs 77% Logrank test p = 0.95) (75% vs 77% Logrank test p = 0.95).

No different EFS was detect between CIG and DIG patients obtained CMR (75% vs 77% Logrank test p = 0.95) (Fig. 7).

As summarized in Table 3 considering the CT-Based Criteria of the Lugano Classification 20 CRs and 30 disease persistence were found ad EoT ceCT.

**Table 3:** EoT response in agreement with CT-based criteria.

CT-criteria	CIG	DIG
CR	20	–
PR	5	9
SD	2	3
PD	10	1

EoT imaging results provided by either PET or ceCT did not predict overall survival (Log-rank test p = 0.11, and p = 0.3), respectively. Similarly, no significant association between cases with additional nodal lesions on ceCT and initial grading and FLIPI score was found (Chi square p > 0.05).

Finally, the inter-rater reliability was excellent with a very high Cohen’s Kappa of 0.879 and overall concordance of 0.955, respectively.

## Discussion

Hybrid imaging has greatly expanded the oncology diagnostics due to the possibility to obtain different information (morphology, structure, metabolism, proliferation) in a one-stop-shop diagnostic procedure. The addition of ceCT to conventional PET/ldCT is useful in some settings as surveillance of high risk colorectal cancer and ovarian cancer, diagnosis of recurrent pancreatic cancer, liver metastases and peritoneal and retroperitoneal lesions^16–19^. Indeed, the role of a PET/contrast-enhanced CT is still a controversial topic in evaluation of lymphoproliferative disease^11,20,21^. PET/CT is generally used to assess response in FDG-avid histologies using PET-based response criteria (5-point scale)^22,23^. Unenhanced PET/CT and PET/ceCT were compared in patients with aggressive lymphoma without statistically significant differences in the number of detected nodal and extra-nodal sites, concluding that in some cases the disease may be upstaged by the addition of ceCT^24–26^. On the other hand PET/ceCT could play a similar role in the detection of residual disease in FL although PET/CT is more sensitive and specific than ceCT for detecting residual disease^12,13,27^.

Follicular lymphomas present relevant differences in FDG uptake that correlate with the histologic grade, tumor aggressiveness and patients’ prognosis^28^. Due to FL heterogeneity and considering the frequency of residual disease after treatment, the choice of imaging procedure for EoT assessment is still a broadly debated topic, especially in patients with prevalent abdominal involvement that generally led inferior outcome^29,30^. Several clinical conditions, in combination with tumor behavior, could affect the clinical status thus complicating and limiting the use of conventional imaging modality in these patients. Several studies have already documented PET/CT as a sensitive, specific and accurate imaging modality for the assessment of treatment response and good predictor of patient outcome in FL^31–33^. Irrespectively of grading, Trotman et al. confirmed these findings after the introduction of first-line immunochemotherapy emphasizing that PET/CT could be considered a standard for therapy response assessment. Applying Deauville 5-point scale evaluation with a cut-off ≤ 3, generally used for HL and DLBCL response assessment, the role of PET/CT- based outcome prediction after first-line immuno-chemotherapy for FL was reinforced^34^. Kostakoglu et al. presented results of a post induction therapy in 75 patients with FL, highlighting that variations of different PET parameters were associated with PFS and response^35^. Our current data showed high diagnostic accuracy of PET/ldCT protocol in response assessment. A limited number of lymphadenopathies were reported following the CT-based response criteria in the mesenteric, retroperitoneal and pelvic area, in addition to PET/ldCT findings, all classified as FDG-negative (i.e. uptake less than liver activity DS ≤ 4). Our results confirmed previous data reported by Morimoto et al. that underlined a limited increase of diagnostic accuracy evaluating nodal status of retroperitoneal and pelvic lymphatic pathways using PET/ceCT in malignant lymphoma^36^. In our analysis, we compared the PET-based versus CT-based response criteria included in the Lugano classification and recorded only 13 cases of discordant assessment. 77% (10/13) of these patients presented a persistent CMR during FU in accord with Deauville score assessment, pointing out a comparable relapse rate between CIG and DIG subgroups. In addition, all our patients underwent a maintenance regimen with rituximab, following the PRIMA study scheme. This approach, in accordance with data previously published supporting a significant long term PFS in patients treated with Rituximab maintenance after immune-chemotherapy. PET/ldCT was a better predictor of EFS than ceCT showing also higher PPV (65% vs. 46%), confirming an irrelevant impact of the additional findings detected by ceCT. In particular, our data support the value of metabolic response evaluation even in indolent disease such as FL, in spite of a higher anatomical detail offered by ceCT, suggesting PET/ldCT as an accurate end-of-treatment imaging procedure.

The small number of the patients enrolled is the main limitation of our study. The strict criteria of selection including a fixed imaging study protocol and immune-chemotherapeutic approach limited the number of eligible patients but, on the other hand, allowed to select a homogeneous population of FL representative of a clear clinical setting. In fact, in this cohort we observed, the 38% of persistent or relapsed disease after standard immune-chemotherapy, in accordance with data reported in literature. Although our preliminary results need to be confirmed in larger prospective cohorts we think our explorative experience may be a solid base to design the following studies.

In conclusion, our results indicate that the additional clinical impact of ceCT to PET imaging in assessing end-of-treatment response in FL is limited, confirming the PET/ldCT as the modality of choice and suggesting to limit the acquisition of additional ceCT images only for doubtful cases of residual disease in mesenteric and pelvic area. This diagnostic approach would be less expensive, minimize diagnostic radiation exposure and preserve renal function.

## Methods

50 consecutive patients (19 female and 31 males; mean age 63 ± 10 years; range 45–83) with new confirmed diagnosis of FL were enrolled between January 2015 and December 2017. The null hypothesis was draft through the results of a preliminary study (https://ashpublications.org/blood/article/128/22/5925/95059/Limited-Benefit-of-Additional-Contrast-Enhanced-CT). Minimum sample size was specified using following parameters:

- Primary outcome: area under the curve
- Null hypothesis: AUC PET/ceCT = AUC PET/ldCT = 0.80
- Alternative hypothesis: PET/ceCT = 0.95
- α = 0.05
- Power = 1 − β = 0.80
- N = 50 patients

Our series was characterized by prevalent nodal and extra-nodal involvement in abdomen and pelvis without bulky masses at staging. All were homogenously treated with standard immune-chemotherapy including monoclonal antibody drug (Rituximab) + chemotherapy [i.e. cyclophosphamide-hydroxydaunorubicin-oncovin-prednisone (CHOP) in 27, Bendamustine in 20 and Chlorambucil in 3 cases respectively]. All patients received additional maintenance therapy with rituximab, following the PRIMA study scheme^37,38^. PET/ldCT was carried out 3–4 weeks after the last treatment. A contrast-enhanced CT (ceCT) examination, as a part of standard diagnostic work-up, was co-registered to PET/ldCT within the same imaging session. The two CT scans were performed consecutively in all patients. Clinical data including sex, age, histological grade, renal function and Follicular Lymphoma International Prognostic Index (FLIPI) were recorded. Exclusion criteria were previous non-FL cancers treated with chemotherapy and/or radiotherapy. Patients were monitored for at least 36 months (median FU of 46 months, range 36–60) to calculate event-free survival (EFS). Follow-up (FU) consisted in clinical history collection and physical examination every 3–6 months for 2 years, and every 6–12 months subsequently. Complete blood count (CBC) and routine clinical chemistry were obtained every 6 months for 2 years, then only as needed at judgement of the attending physician. Imaging examination (minimal adequate radiological) were performed every 6 months for 2 years, annually up to 5 years (optional) and in any case of clinical suspicion of disease recurrence^39^.

All procedures performed in studies involving human participants were in accordance with the ethical standards of the institutional and/or national research committee and with the 1964 Helsinki Declaration and its later amendments or comparable ethical standards. The experimental protocol was approved by the Institutional Review Board of Imaging Institute of Southern Switzerland (IIMSI) and Oncology Institute of Southern Switzerland (IOSI) at Ente Ospedaliero Cantonale (EOC). The requirement for informed consent was waived by the Advisory Research Board and the Ethic Committee of Canton Tessin, Switzerland.

### PET/CT protocols

PET/CT examinations were performed on full-ring integrated PET/CT systems (Biograph mCT 40). CT scans obtained with a low-dose protocol were used for attenuation-correction of the PET images. All patients were fasting for at least 6 h before the injection of 210 to 370 MBq (3 MBq/kg) 18FDG. Blood glucose measured before injection of the radiotracer was < 160 mg/dL in all patients. PET data were acquired in 3-dimensional mode from the mid-thigh toward the base of the skull after a standardized uptake time of 60 min^40–42^. The PET acquisition time was at least 2.5 min per bed position. Images were reconstructed with validated and commercially available iterative algorithms, and standardized uptake values (SUVmax) were automatically calculated. Following PET/ldCT protocol a contrast-enhanced CT (ceCT) of the head-neck, thorax, abdomen and pelvis was performed in all patients. The scanning parameters were as follows: section thickness, 3 mm; voltage, 100 kV; tube current, 150 mA; and matrix 500 × 500. An intravenous bolus dose of 90 mL of nonionic iodinated contrast agent (Accupaque, GE healthcare) was administered at a rate of 2 mL/s.

### Interpretation criteria

Two different observers (one board certified nuclear medicine physician and 1 board certified radiologist with more than 15 years of experience in PET/CT imaging each) evaluated PET/ldCT and, respectively, PET/ceCT images. They were blinded to demographic, pathological and clinical data. The 18FDG-PET/CT images were analyzed following a standard protocol on a dedicated workstation (Siemens SyngoVia^®^ workstation; Siemens, Erlangen, Germany). Dedicated software (MM-oncology, Syngo^®^) automatically estimated the average and maximum standardized uptake value (SUV) (SUVmean and SUVmax) of each lesions using a semi-automated isocontouring 3D VOI tool. Number and sites of nodal and extra-nodal FDG avid-lesion were noted and compared with the background activity, mediastinal blood-pool and liver uptake and graded from score 1 to 5 applying standard 5-point scale and Deauville criteria^43^. According to the Lugano classification a Deauville score of 4 and 5 identifies residual metabolic disease. Stable disease (SD), partial response (PR) or progressive disease (PD) were defined comparing EoT PET results to the PET baseline findings^44^. Additionally, the radiologist also recorded pathological findings detected in lymph nodes and extra nodal tissue on PET/ceCT images following the CT-Based response criteria of the Lugano Classification. Patients were classified as having PET and ce-CT matched response (concordant imaging group, CIG) or PET and ce-CT unmatched response with additional residual morphological disease on ceCT (discordant imaging group, DIG). Relapse rates and Event-Free Survival (EFS) were compared between CIG and DIG patients.

### Statistics

The PET/ldCT and PET/ceCT results were compared either on per patient or per lesion basis. Differences between categorical data obtained by PET/CT and ce-CT on a per-patient basis were analyzed using χ^2^ statistics. Concordance between PET reading and radiological reading on a per-station basis (nodal/extranodal) was assessed using kappa statistics.

EFS was defined according to the revised NCI criteria^45^ and estimated by the Kaplan–Meier method, and patient groups were compared by the log-rank test. For all test a p value < 0.05 was considered statistically significant. Statistical analysis was performed using MedCalc^®^ Software package 2016 for Windows.

### Ethics approval

All procedures performed in studies involving human participants were in accordance with the ethical standards of the institutional and/or national research committee and with the 1964 Helsinki Declaration and its later amendments or comparable ethical standards. The experimental protocol was approved by the Institutional Review Board of Imaging Institute of Southern Switzerland (IIMSI) and Oncology Institute of Southern Switzerland (IOSI) at Ente Ospedaliero Cantonale (EOC). The requirement for informed consent was waived by the Advisory Research Board and the Ethic Committee of Canton Tessin, Switzerland.

## Data Availability

The datasets generated and/or analyzed during current study are available from the corresponding author on reasonable request.
